# A UV‐C LED‐based unit for continuous decontamination of the sheath fluid in a flow‐cytometric cell sorter

**DOI:** 10.1002/elsc.202200025

**Published:** 2022-07-08

**Authors:** Jenny Kirsch, Kerstin Heinrich, Daniel Kage, Johannes Glaab, Benjamin Grothe, Konrad v. Volkmann, Toralf Kaiser

**Affiliations:** ^1^ Flow Cytometry Core Facility Deutsches Rheuma‐Forschungszentrumein Institut der Leibniz‐Gemeinschaft Berlin Germany; ^2^ Ferdinand‐Braun‐Institut gGmbH Leibniz‐Institut für Höchstfrequenztechnik Berlin Germany; ^3^ APE Angewandte Physik und Elektronik GmbH Berlin Germany

**Keywords:** aseptic sorting, flow‐cytometric cell sorting, UV‐C LEDs

## Abstract

Aseptic cell sorting is challenging, especially when a flow‐cytometric cell sorter is not operated in a sterile environment. The sheath fluid system of a cell sorter may be contaminated with germs such as bacteria, yeasts, viruses, or fungi. Thus, a regular chemical cleaning procedure is required to prepare a sorter for aseptic cell sorting by flushing the fluidic system. However, this procedure is time consuming, and most importantly, the researcher can never be sure that the cleaning process was successful. Here we present a method in which the sheath fluid of a cell sorter was decontaminated by irradiation with UV‐C light using a flow‐through principle. Using this principle, we were able to achieve a 5 log reduction of bacteria in the sheath fluid.

**Abbreviations**
LB: lysogeny broth
LED: light‐emitting diode
*P. aeruginosa*
: *Pseudomonas aeruginosa*
PI: propidium iodide
UV: ultraviolet

## 1 INTRODUCTION

Flow‐cytometric cell sorting is a key technology in bio‐medical research laboratories used to separate cells based on their biological and physical properties [1]. The contamination of sorted cells with microorganisms such as bacteria, yeasts, viruses, or fungi by the cell sorter must be avoided to enable cell cultivation or to prevent misleading subsequent experimental results.

Flow‐cytometric cell sorters typically do not operate within a sterile environment, making contamination with microorganisms very likely, for example, when the sheath fluid reservoir is opened for refilling. Thus, a chemical cleaning procedure based on sodium hypochlorite or ethanol is required to prepare a cell sorter for aseptic cell sorting [2]. Such a procedure is time consuming and, more important, the operator can never be sure if the cleaning process was successful [3]. Furthermore, residues of cleaning reagents in the fluidics can cause harms to sorted cells due to toxicity.

In many cell sorting laboratories, antibiotics are added to the collection medium of the sorted cells to prevent the proliferation of bacteria entering through the sheath fluid. This, in turn, may lead to an unwanted change in gene expression and regulatory level of the cultured cells and affect the subsequent results [2]. Furthermore, the widespread use of antibiotics promotes the development of resistant bacteria [4, 5].

The lethal effect of UV light [6] is well known for inactivating microorganisms. UV light is divided into the UV‐A (380–315 nm), UV‐B (315–280 nm) and UV‐C (280–200 nm) spectral region.

The antimicrobial effect of UV‐C light is based on the absorption of photons in the wavelength range of 200–280 nm by the DNA, resulting in the formation of pyrimidine dimers, which inhibit DNA replication and consequently block transcription into RNA [7]. UV‐C light can be generated by mercury‐based lamps, pulsed xenon lamps, or more recently by UV‐C LEDs. The latter have very small dimensions and require low current to provide UV‐C doses sufficient to reduce the quantity of bacteria in water by several log10 levels [8]. These properties make UV‐C LEDs very interesting for the decontamination of sheath fluids in flow‐cytometric cell sorters as an alternative to cleaning procedures based on chemical decontamination.

Here we present a method based on a UV‐C unit for flow‐through irradiation of sheath fluid to enable aseptic cell sorting. In a proof‐of‐principle study, the UV‐C unit was placed between the sheath fluid tank and the nozzle in a BD Influx™ cell sorter (see supplementary data for detailed description). The decontamination efficiency of the unit was tested on bacteria obtained from the laboratory's room air.

## 2 METHODS & RESULTS

**FIGURE 1:**
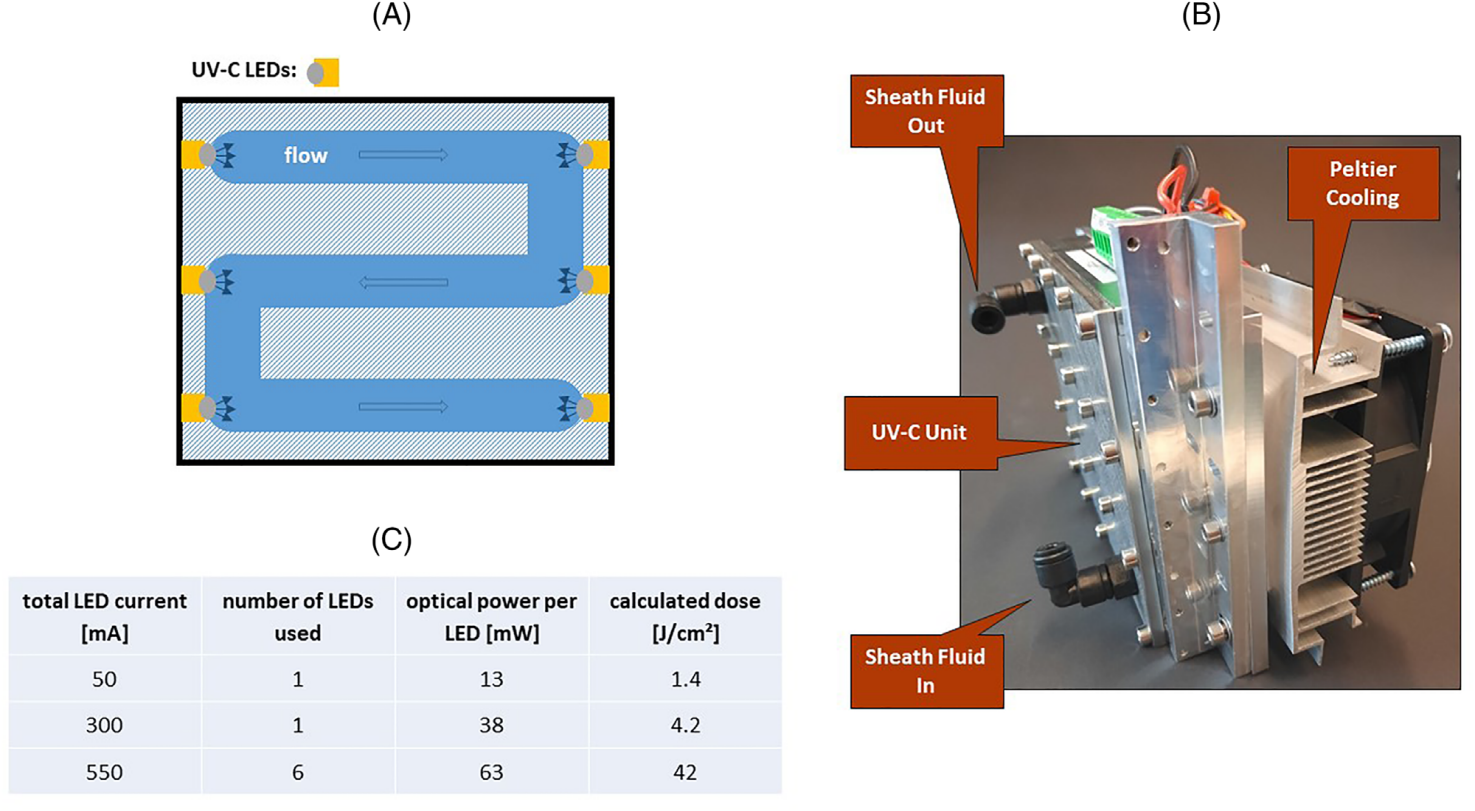
(A) Top view scheme of the UV‐C LED unit. The unit has a total volume of 60 ml and is irradiated by six UV‐C LEDs. This results in a maximum total UV‐C dose of 42 J/cm^2^ at a sheath fluid pressure of 18 psi and an LED current of 550 mA. The channel cross section is 16 × 10 mm. (B) Picture of the unit including the Peltier cooling device. The dimension of the overall unit is 120 × 140 × 90 mm. (C) UV‐C LED parameters to generate three different UV‐C doses. The residence time of the sheath fluid inside the unit was 10 min for all doses at a sheath fluid pressure of 18 psi. The optical power per LED was taken from the data sheet

The UV‐C unit (APE, Berlin) consists of a meandering stainless steel fluid path with a total volume of 60 ml in which the sheath fluid is irradiated by six UV‐C LEDs (Luminus, XST‐3535‐UV‐A60‐CE275‐00) with an emission wavelength of 275 nm. The LEDs are located at each channel turning point (Figure 1A). The total UV‐C dose applied to the sheath fluid passing through the unit is given by the LED current, the resulting LED radiation flux, the number of LEDs, and the residence time of the sheath fluid inside the unit. Three different UV‐C doses were applied by varying the LED current and the number of active LEDs while keeping the sheath fluid pressure constant (Figure 1C). A coarse estimate of the expected dose was obtained from a simplified calculation and a detailed description of the estimation of the UV‐C dose can be found in the supplement. The calculation assumes a transmission of UV radiation as in free space. However, due to light absorption and turbulences in the fluid path, the real UV‐C dose is very likely less than the calculated dose.

**PRACTICAL APPLICATION**

The sheath fluid of a flow‐cytometric cell sorter can potentially be contaminated with proliferating microorganisms. Our approach uses UV‐C light emitting diodes (LEDs) to irradiate the sheath fluid of a cell sorter in process. This procedure prevents the effects of bacterial contamination on the cell culture of the sorted cells.

The unit is equipped with an active Peltier cooling element (Figure 1B) to prevent heating of the sheath fluid due to the power dissipation of the UV‐C LEDs. Temperature variations could lead to variations in the position of the point where the sheath fluid jet breaks into droplets (break‐off point) due to changes in the viscosity and density of the sheath fluid, which in turn can affect purity and yield of the sorted cells [9]. The sheath fluid temperature set point was 15°C during all experiments.

The efficiency of the UV‐C unit to inactivate microorganisms was characterized using flow cytometry, solid culture medium (LB agar), and the cultivability of Jurkat cells sorted in contaminated and irradiated sheath fluid. To this end, the sheath fluid of a BD Influx™ cell sorter was contaminated with *P. aeruginosa* at a concentration of 1.1 × 10^5^ bacteria/ml.

**FIGURE 2:**
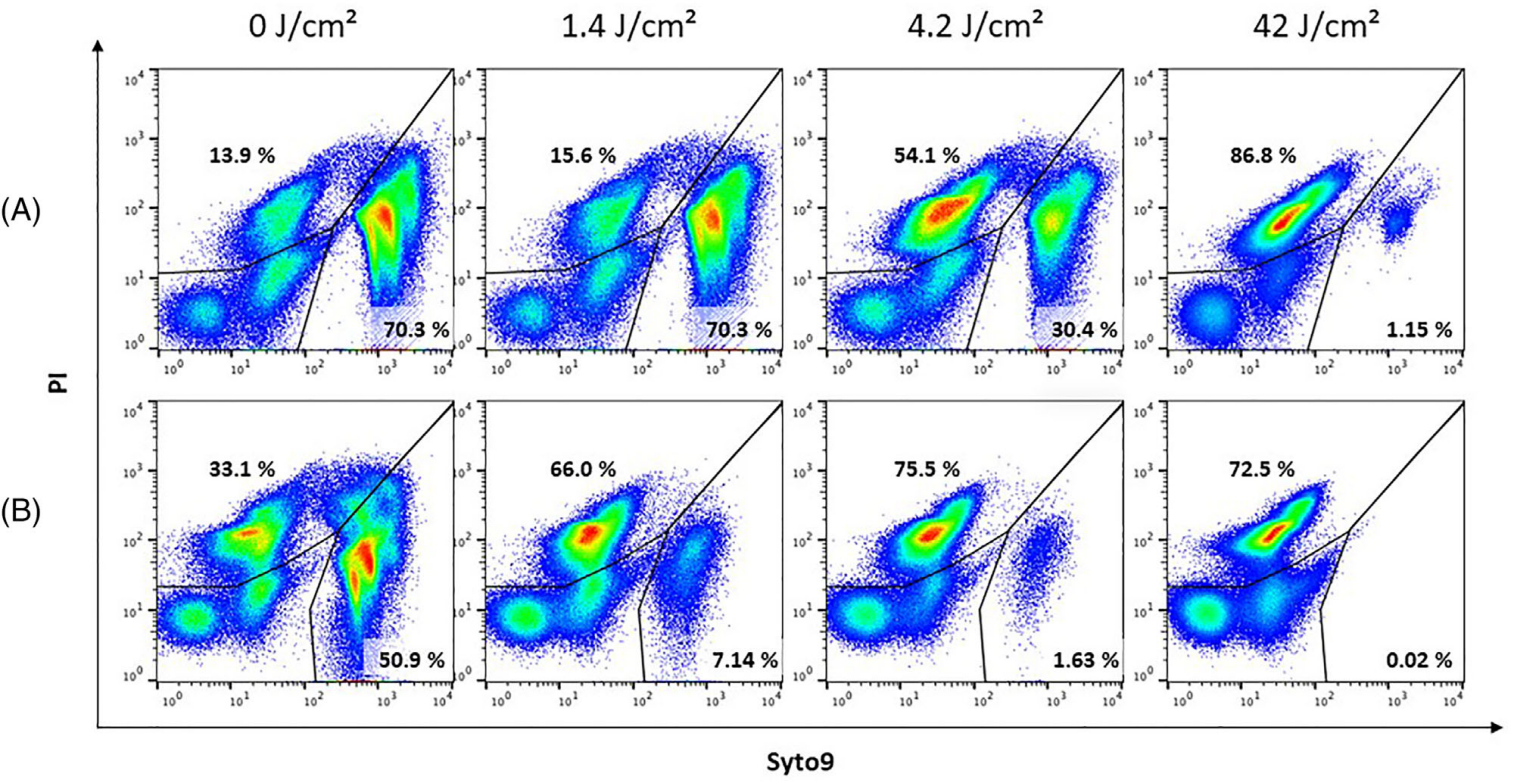
Flow‐cytometric analysis of *P. aeruginosa* to define the efficiency of the UV‐C unit. The sheath fluid of a cell sorter was contaminated with *P. aeruginosa* and irradiated with four different UV‐C doses (0, 1.4, 4.2, 42 J/cm^2^). The irradiated and non‐irradiated sheath fluid was collected and stained with nucleic acid dyes Syto9 and PI to analyze the membrane integrity of the bacterial cells. Samples were analyzed (A) immediately or (B) 24 h after irradiation. With increasing UV dose, the relative size of the Syto9‐positive population decreased from 70.3% to 1.15% for directly stained samples and from 50.9% to 0.02% for staining 24 h after irradiation. The events in the lower left gate are the cytometer's background and particles that cannot be stained with nucleic acid dyes

The results of the flow‐cytometric analysis using the nucleic acid dyes Syto9 and PI are shown in Figure 2. The resulting fluorescence patterns reflect the different binding capacity of Syto9 and PI to the nucleic acids in dependence of the applied UV‐C dose and the time of analysis (Figure 2A directly after irradiation, Figure 2B 24 h after irradiation). The relative size of the Syto9‐positive population decreases from 70.3% to 1.15% with increasing UV‐C dose from 0 to 42 J/cm^2^ (Figure 2A). However, if the samples were cultivated for 24 h after irradiation and subsequently stained, the relative size of the Syto9‐positive population decreases from 50.9% to 0.02% in the UV‐C irradiated sample (Figure 2B). This suggests that (a) the outer membrane of the gram‐negative *P. aeruginosa* is increasingly damaged with increasing UV‐C dose [10, 11, 12] and (b) the damaging effects of UV‐C light is not fully detectable immediately after irradiation. This possibly indicates that repair mechanisms are not active or not efficient enough to enable bacterial growth after UV‐C irradiation.

The flow‐cytometric results were confirmed by plating the irradiated and non‐irradiated sheath fluid on LB agar. For all doses applied, no bacterial colonies were detected after 24 h of cultivation. Consequently, using the UV‐C LED unit in the described configuration, a 5 log10 reduction of *P. aeruginosa* could be achieved for all applied doses which meets the effectiveness of the reduction levels of the chemical washing protocols suggested by the cytometer manufacturer [2].

Jurkat cells were sorted in sheath fluid contaminated with *P. aeruginosa* to assess the impact of UV‐C irradiation of sheath fluid on cell cultivability. After sorting, the cells could be cultured for at least 72 h even when applying the lowest UV‐C dose of 1.4 J/cm^2^. However, if the sheath fluid is not irradiated by UV‐C light, the cell culture was overgrown with bacteria within 24 h of incubation. This confirmed the results determined by plating experiments on LB agar where the lowest UV‐C dose of 1.4 J/cm^2^ was sufficient to prevent proliferation even if the membrane was not completely degraded as indicated by nucleic acids staining (Figure 2).

## 3 DISCUSSION

Following the calculated dose estimates, the UV‐C unit enables a maximum dose of 42 J/cm^2^, which is about 5400 times above the required dose to achieve a 4 log10 reduction of *P. aeruginosa*. Thus, even if the applied UV‐C dose is lower than the calculated dose, it is very likely that other microorganisms, such as yeasts, fungi or viruses [6, 11] can also be successfully inactivated by the suggested method. However, additional LEDs can be added to the unit if a higher dose is required. In our experience, most of the contamination is caused in the sheath fluid tank. Thus, the use of UV‐C LEDs inside the tank would also be conceivable, but technically more complex. Overall, the presented UV‐unit is able to effectively decontaminate the sheath fluid in a flow‐cytometric cell sorter, allowing cell sorting without the need for antibiotics in subsequent cell culturing. Moreover, it helps to avoid extensive chemical cleaning of the cell sorter tubing. It is therefore a valuable method to perform cell sorting for studies that rely on subsequent culture of sorted cells in an antibiotic‐free environment.

## CONFLICT OF INTEREST

K.v.V and B.G. are employees of APE. All other authors declare no commercial or financial conflict of interest.

## Supporting information

SUPPORTING INFORMATION

## References

[1] Cossarizza A , Chang HD , Radbruch A , et al. Guidelines for the use of flow cytometry and cell sorting in immunological studies (second edition). Eur J Immunol. 2019;49(10):1457‐1973. 10.1002/eji.201970107 31633216 PMC7350392

[2] McIntyre CA , McCord R , Vrane D , Decontamination of the BD FACSAria II or BD FACSAria III system using the prepare for aseptic sort procedure. 2010;p8.

[3] Muraki Y , Hongo S , Ohara Y . Contamination of the cell sorter fluidics system with the water‐borne bacterium Burkholderia cepacia. Cytometry Part A. 2012;81A(2):105‐107. 10.1002/cyto.a.21142 21905213

[4] Ryu AH , Eckalbar WL , Kreimer A , Yosef N , Ahituv N . Use antibiotics in cell culture with caution: genome‐wide identification of antibiotic‐induced changes in gene expression and regulation. Sci Rep. 2017;7(1):7533. 10.1038/s41598-017-07757-w 28790348 PMC5548911

[5] Subedi D , Vijay AK , Willcox M . Overview of mechanisms of antibiotic resistance in Pseudomonas aeruginosa: an ocular perspective. Clin Exp Optom. 2018;101(2):162‐171. 10.1111/cxo.12621 29044738

[6] Hijnen WaM , Beerendonk EF , Medema GJ . Inactivation credit of UV radiation for viruses, bacteria and protozoan (oo)cysts in water: a review. Water Res. 2006;40(1):3‐22. 10.1016/j.watres.2005.10.030 16386286

[7] Pfeifer GP . Formation and processing of UV photoproducts: effects of DNA sequence and chromatin environment. Photochem Photobiol. 1997;65(2):270‐283. 10.1111/j.1751-1097.1997.tb08560.x 9066304

[8] Würtele MA , Kolbe T , Lipsz M , et al. Application of GaN‐based ultraviolet‐C light emitting diodes – UV LEDs – for water disinfection. Water Res. 2011;45(3):1481‐1489. 10.1016/j.watres.2010.11.015 21115187

[9] Petersen TW , van den Engh G . Stability of the breakoff point in a high‐speed cell sorter. Cytometry Part A. 2003;56A(2):63‐70. 10.1002/cyto.a.10090 14608633

[10] Zhang S , Ye C , Lin H , Lv L , Yu X . UV disinfection induces a Vbnc state in escherichia coli and pseudomonas aeruginosa. Environ Sci Technol. 2015;49(3):1721‐1728. 10.1021/es505211e 25584685

[11] Song K , Mohseni M , Taghipour F . Application of ultraviolet light‐emitting diodes (UV‐LEDs) for water disinfection: a review. Water Res. 2016;94:341‐349. 10.1016/j.watres.2016.03.003 26971809

[12] Berney M , Hammes F , Bosshard F , Weilenmann H‐U , Egli T . Assessment and interpretation of bacterial viability by using the LIVE/DEAD BacLight kit in combination with flow cytometry. Appl Environ Microbiol. 2007;73(10):3283‐3290. 10.1128/AEM.02750-06 17384309 PMC1907116

