# Supplementary material for: A UV‐C LED‐based unit for continuous decontamination of the sheath fluid in a flow‐cytometric cell sorter
Source: Eng Life Sci. 2022 Jul 8;22(8):550–3. doi: 10.1002/elsc.202200025 (PMC9349136; doi:10.1002/elsc.202200025)
Supplement: Supplementary file 1 — SUPPORTING INFORMATION [file ELSC-22-550-s001.docx]

**Supporting Information**

**Kirsch et al.**

**A UV-C LED-based unit for continuous decontamination of the sheath fluid in a flow-cytometric cell sorter**

**Methods**

**Preparation of bacterial stock**

An aliquot of 50 µl bacterial contaminated sheath fluid (PBS - Phosphate buffered saline) was plated on LB agar plates (MP Biomedicals™ LB -agar, Lennox) and grown overnight at 37 °C. Bacteria were then identified by an independent laboratory as gram-negative *P. aeruginosa*.

A glycerol stock was prepared to use the *P. aeruginosa* as a sheath fluid contamination source for all subsequent UV-C irradiation experiments. Briefly, 50 µl PBS from the contaminated sheath fluid reservoir were plated on LB agar and grown overnight at 37 °C. A single clone was picked, inoculated into 2 ml LB medium and grown with agitation overnight at 37 °C. This bacterial suspension was centrifuged for 5 min at 1000 × g, the resulting pellet was resuspended in 50% (v/v) LB medium:50% (v/v) glycerol and stored at -80 °C. For the irradiation experiments, 5 µl of the stock solution was added to 2 ml of LB medium and grown overnight at 37 °C to prepare the sheath fluid reservoir by inoculation.

**Calculation of UV doses**

The UV dose is the product of intensity and illumination time. Further, the light intensity depends on the distance from the LED and the reflective properties of the channel walls. In free space, a reasonable approximation is that the emitted light distributes homogeneously over the basal area of a cone. Then, the intensity $I_{f}$ at a distance $d$ from an LED with an optical output power $P$ and an opening angle of the light cone $\vartheta$ is

| $I_{f}(d)=\frac{P}{\pi d^{2}tan^{2}(\vartheta/2)}$. | (1) |
| --- | --- |

Within the channel (width/height $w$ and $h$) of the UV module, the light cone intersects the channel walls at a certain point. Two limiting cases are considered for the sake of simplicity: (a) the walls absorb all of the impinging light and (b) the channel walls reflect all of the impinging light. In case (a), the intensity in the channel $I_{c}$ follows Eq. (1). In case (b), it first follows Eq. (1), then reaches the region where part of the light is reflected by the walls and finally the intensity takes a constant value $\frac{P}{w\cdot h}$ once the light cone completely covers the channel cross section. The dose $D$ absorbed by a particle accumulates travelling through the channel depends on the flow speed $v$ and the local light intensity:

| $D = \int_{x_{1}}^{x_{2}} I_{c}\left( x \right)\frac{dx}{v}\approx\sum_{j} I\left( x_{j} \right)\frac{\Delta x}{v}$, | (2) |
| --- | --- |

Where the particle travels from point $x_{1}$ to $x_{2}$ through the channel. Case (b) was used for dose calculations. Further optical UV-C light absorption by the sheath fluid and turbulences in the flow channel are not considered. The calculations were performed with an Octave script. A thorough description can be found with the example code [1].

**Preparation of the BD Influx**™ **cell sorter**

The UV-C reactor was installed near the nozzle head of a BD Influx™ cell sorter (Fig. 1). Thus the decontamination of the sheath fluid takes place right before cells are injected into the sheath fluid stream. The cells to be sorted are not irradiated with UV-C light.

Initially, the entire fluidics of the BD Influx™, including the reactor channel, was cleaned with BD FACS Clean for 20 min (this corresponds to an double exchange of the fluidic volume in the reactor) followed by flushing with DI water for 40 min to avoid contamination caused by the sheath fluid. The 0.2µm sheath fluid filter of the cell sorter was removed from the sheath fluid line to allow high contamination of the entire sheath fluid system.

Finally, the cell sorter sheath fluid reservoir was filled with bacterial contaminated sheath fluid in a concentration of 1.1x10^5^ bacteria/ml as determined by BD Trucount^TM^.


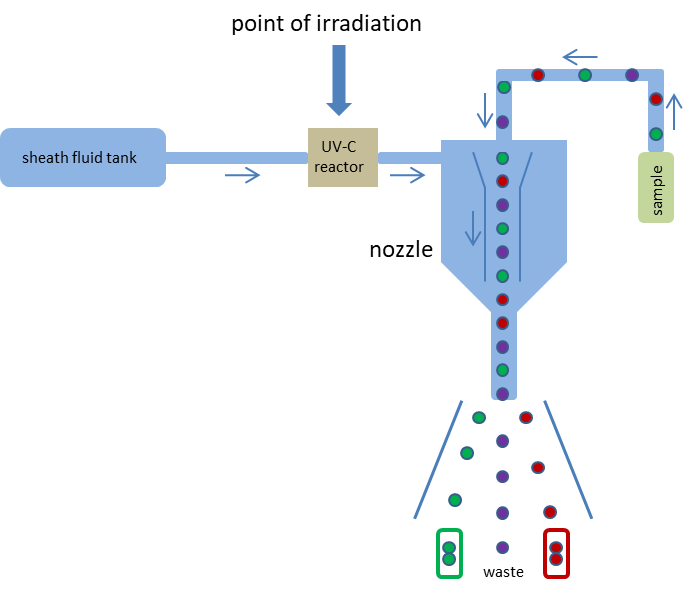


Figure 1. Schematic view of the setup of the UV-C unit installed in a BD Influx™ cell sorter. The unit was placed at a short distance to the nozzle head between sheath fluid reservoir and nozzle. Cells from the sample are not irradiated by UV-C light.

**UV-C irradiation of contaminated sheath fluid**

Irradiation of the contaminated sheath fluid proceeded beginning with the highest irradiation dose 42 J/cm^2^), followed by the next lower doses (4.2 J/cm^2^, 1.4 J/cm^2^), and concluded with no irradiation. All sheath fluid samples were taken from the outlet of the nozzle (2 ml each). After each radiation dose the system was running for 20 min to exchange the reactor volume twice to ensure that the bacteria are irradiated with a homogeneous dose.

After irradiation experiments, the contaminated fluidics and the sheath fluid reservoir were rinsed with BD FACS Clean followed by DI water in order to prepare the sorter for the subsequent analysis of the irradiated samples.

**Cultivation of bacteria**

The irradiated and non-irradiated sheath fluid samples were plated on antibiotic-free lysogeny broth (LB) agar (sample volume 50 µl) and incubated in duplicates at 37°C for 24 h immediately after collection.

**DNA Staining**

The cell physiology of the bacteria as a function of irradiation dose was determined by flow cytometry using the LIVE/DEAD® BacLight™ Viability Kit (Thermo Fisher Scientific, Waltham, USA) based on the nucleic acid dyes propidium iodide (PI) and Syto9. Syto9 binds the DNA/RNA of all cells, while PI only binds DNA/RNA of cells having a disrupted cytoplasmic membrane as a consequence of UV-C irradiation. The uptake of PI into cells with damaged cytoplasmic membrane leads also to a reduction of Syto9 fluorescence by FRET [2].

Syto9 and PI are excited by laser light of 488 nm and detected with 530/30 and 660/20 band pass filters, respectively. The measurements were performed on the BD Influx™ using a PMT for forward scatter (FSC) detection allowing small particle detection.

The collected sheath fluid samples were stained and analyzed immediately after irradiation and after 24 h of incubation at 37°C. This ensures that the time depended damaging effects of UV-C light is considered. The non-irradiated sample was treated in the same manner but stored at 4°C between the time points of analysis to prevent bacterial overgrowing.

The staining was conducted as follows: 500 µl of irradiated and non-irradiated samples were added

to a BD Trucount™ Tube containing a fixed number of beads, stained with 0.75 µl of 3.34 mM Syto9

and 0.75 µl of 20 mM PI and incubated at room temperature in the dark for 1 h. Finally, 20,000 bead

events were acquired on the cell sorter, corresponding to at least 100,000 bacterial events from each

sample. Data analysis was performed with FlowJo 10 (FlowJo, BD, USA).

**Jurkat cell sort in irradiated and non-irradiated sheath fluid**

Jurkat cells were sorted in sheath fluid contaminated with *P. aeruginosa* (concentration of 8.8x10^5^ bacteria/ml) to determine whether UV-C irradiation of the sheath fluid would enable the cultivation of the sorted Jurkat cells (DSMZ). Before sorting, the cells were washed once and resuspended in PBS (Th. Geyer, Germany). Four samples (each 2.5×10^5^ cells) with increasing irradiation doses (42, 4.2, 1.4, 0 J/cm^2^) were sorted and collected in Falcon tubes coated with PBS/BSA. The sorted cells were cultivated in RPMI-1640/Glutamax (Gibco) medium, supplemented with 10% FBS (fetal bovine serum, Corning), without addition of antibiotics, in an atmosphere of 5% CO_2_, 95% air and 95% humidity at 37°C. After 24 and 72 h, the cell culture suspensions were checked by microscope to identify bacterial contamination [3].

**Temperature stabilization of the UV-C reactor**

Fluctuations in the temperature of the sheath fluid can lead to fluctuations in the break-off time and break-off position which in turn affect the sorting purity and recovery [4], [5]. Therefore, the module is equipped with an active cooling system, which prevent heating of the sheath fluid due to the power dissipation of the UV-C LEDs.

The impact of the sheath fluid temperature on the break-off point was determined by stepwise increasing the sheath fluid temperature from 14 to 22°C while measuring the required drop drive amplitude to keep the break-off point in a constant position. As shown by Figure 2 the amplitude of the drop drive must be changed by 0.28 V/K to compensate for the temperature fluctuations in order to keep the break-off point in a constant position. Hence, a constant module temperature set point of 15 °C was chosen for all experiments to avoid fluctuations of the break-off point or temperature-induced cell damage.


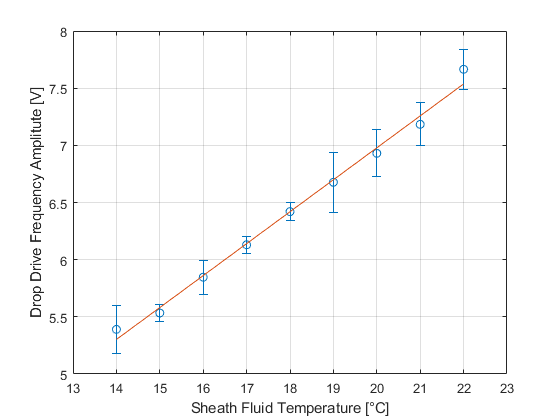


Figure 2. The piezo amplitude required to keep the break-off point in a stable position is correlated to the temperature of the sheath fluid by 0.28 V/K.

**References**

[1] *10.5281/zenodo.5512394*. [Online]. Available: doi.org/10.5281/zenodo.5512394

[2] S. M. Stocks, “Mechanism and use of the commercially available viability stain, BacLight,” *Cytometry Part A*, vol. 61A, no. 2, pp. 189–195, 2004, doi: 10.1002/cyto.a.20069.

[3] A. Mirjalili, E. Parmoor, S. Moradi Bidhendi, and B. Sarkari, “Microbial contamination of cell cultures: A 2 years study,” *Biologicals*, vol. 33, no. 2, pp. 81–85, Jun. 2005, doi: 10.1016/j.biologicals.2005.01.004.

[4] Toralf Kaiser, Katharina Raba, Alexander Scheffold, and Andreas Radbruch, “A sheath-cooling system to stabilize side-streams and drop delay during long-term sorts for FACS- Aria® cell-sorter,” 2006, doi: 10.13140/RG.2.1.2755.3681.

[5] T. W. Petersen and G. van den Engh, “Stability of the breakoff point in a high-speed cell sorter,” *Cytometry Part A*, vol. 56A, no. 2, pp. 63–70, 2003, doi: 10.1002/cyto.a.10090.
